# Sensitivity and specificity of an algorithm based on medico-administrative data to identify hospitalized patients with major bleeding presenting to an emergency department

**DOI:** 10.1186/s12874-019-0841-6

**Published:** 2019-10-18

**Authors:** Emmanuel Oger, Marie-Anne Botrel, Catherine Juchault, Jacques Bouget

**Affiliations:** 1 Univ Rennes, EA 7449 REPERES [Pharmacoepidemiology and Health Services Research], Rennes, France; 2 Department of Medical Information, CHU Rennes, Rennes, France

**Keywords:** Validation study, ICD-10, Algorithm, Major bleeding

## Abstract

**Background:**

Validation studies on an ICD-10-based algorithm to identify major bleeding events are scarce, and mostly focused on positive predictive values.

**Objective:**

To evaluate the sensitivity and specificity of an ICD-10-based algorithm in adult patients referred to hospital.

**Methods:**

This was a cross-sectional, retrospective analysis. Among all hospital stays of adult patients referred to Rennes University Hospital, France, through the emergency ward in 2014, we identified major bleeding events according to an index test based on a list of ICD-10 diagnoses. As a reference, a two-step process was applied: firstly, a computerized request for electronic health records from the emergency ward, using several hemorrhage-related diagnostic codes and specific emergency therapies so as to discard stays with a very low probability of bleeding; secondly, a chart review of selected records was conducted by a medical expert blinded to the index test results and each hospital stay was classified into one of two exclusive categories: major bleeding or no major bleeding, according to pre-specified criteria.

**Results:**

Out of 16,012 hospital stays, the reference identified 736 major bleeding events and left 15,276 stays considered as without the target condition. The index test identified 637 bleeding events: 293 intracranial hemorrhages, 197 gastrointestinal hemorrhages and 147 other bleeding events. Overall, sensitivity was 65% (95%CI, 62 to 69), and specificity was 99.0%. We observed differential sensitivity and specificity across bleeding types, with the highest values for intracranial hemorrhage. Positive predictive values ranged from 59% for “other” bleeding events, to 71% (95%CI, 65 to 78) for gastrointestinal hemorrhage, and 96% for intracranial hemorrhage.

**Conclusions:**

Low sensitivity and differential measures of accuracy across bleeding types support the need for specific data collection and medical validation rather than using an ICD-10-based algorithm for assessing the incidence of major bleeding.

## Background

A reimbursement claim database enables large cohorts to be set up, providing comprehensive data at a relatively low cost [1]. Their use in pharmacoepidemiology has considerably increased in recent years [2]. In France, numerous studies have been conducted using the French National Health database (SNDS, previously known as SNIIRAM) [3–7]. The French hospital database (PMSI), part of the SNDS, provides a discharge diagnosis (ICD-10 codes) for all patients admitted to hospital in France. It is considered that hospital-based data, and discharge codes in particular, can be used as valuable sources of information to define patient populations, assess comorbidities [8] or the severity of disease, determine patient outcomes [3] and drug effectiveness [4], and detect adverse events, including major bleeding [5–7].

Major bleeding is the most feared serious adverse reaction when using antithrombotic agents. Estimating the occurrence of major bleeding events is therefore a key issue. Patients presenting major bleeding are mostly referred to hospital, which therefore makes hospital-based data useful. Nonetheless, caution is needed regarding the accuracy of codes in hospital-based data for major bleeding event identification: for instance when assigning primary or secondary discharge diagnoses, a focus on the reimbursement of the care delivered could hide the real reason for admission; coding inaccuracies or inconsistencies can occur across care sites, and bleeding events that are not coded could be overlooked; hence a validated algorithm is crucial.

Emergency wards are obviously optimal settings to observe and report serious adverse drug reactions for drugs prescribed in the community. With regard to major bleeding events, the validation of a hospital-based data algorithm could gain from a confrontation with medical charts from emergency wards.

## Methods

### Aim, design and setting

Our objective was to evaluate the sensitivity and specificity of an ICD-10-based algorithm that has already been used [5] to identify major bleeding events in adult patients referred to hospital in a dedicated dataset.

This was a cross-sectional, retrospective analysis conducted at Rennes University Hospital, a tertiary care facility.

### Study population

All hospital stays of adult patients referred to Rennes University Hospital through emergency ward between 01/01/2014 and 12/31/2014 were identified through the hospital registry and were eligible to participate.

### Index test

We used a list of ICD-10 primary hospital discharge diagnosis codes previously published [5]: hospitalization for bleeding, including intracranial (hospital discharge ICD-10 codes I60, I61, I62, S06.3,S06.4, S06.5, S06.6), gastrointestinal (I85.0, K25.0, K25.2, K25.4,K25.6, K26.0, K26.2, K26.4, K26.6, K27.0, K272, K27.4, K27.6,K28.0, K282, K28.4, K28.6, K29.0, K62.5, K92.0, K92.1, K92.2) and other bleeding (D62, N02, R31, R58, H11.3, H35.6, H43.1, H45.0,H92.2, J94.2, K66.1, M25.0, N92.0, N92.1, N92.4, N93.8, N93.9,N95.0, R04.0, R04.1, R04.2, R04.8, R04.9). See Additional file 1 for details on labeling. Each hospital stay was classified into one of four exclusive categories: intracranial, gastrointestinal, other bleeding or no bleeding event. We chose this list because it was derived from and had already been used on French hospital-based data.

### Standard procedure

To provide a reference, a standard two-step process was applied to all hospital stays: firstly, a computerized request for electronic health records from emergency wards using several hemorrhage-related diagnostic codes (see Additional file 2) and specific emergency therapies (red blood cell transfusion, platelet transfusion, vitamin K, protamin sulfate, prothrombin complex concentrate, and FEIBA®, an anti-inhibitor coagulant complex); this request demonstrated good sensitivity (96, 95% exact confidence limits (CL) 80 to 99%) and specificity (100%, exact 95% CL, 99 to 100%) in a pilot study. The probability of bleeding in the discarded records (i.e. records not identified by this request) was consequently considered to be probably very low. Secondly, a review of the selected records was conducted by a medical expert (JB) blinded to index test results, and each hospital stay was classified in one of two exclusive categories: major bleeding or no major bleeding. Major bleeding was defined on at least one of the following criteria in the review of medical charts: unstable hemodynamic (systolic arterial pressure < 90 mmHg or mean arterial pressure < 65 mmHg) or shock, uncontrollable bleeding, need for transfusions or hemostatic procedure (embolization, endoscopic procedure, surgery), and life-threatening locations such as intracranial, intra-spinal, intraocular, retroperitoneal, pericardial, thoracic, intra-articular, intramuscular hematoma with compartment syndrome, acute gastrointestinal bleeding. We considered major bleeding in case of epistaxis when at least two procedures of nasal packing were needed, and in case of hematuria when bleeding continued for more than 12 h despite bladder washing. An extensive chart review was simply unrealistic, because of the expense involved in reviewing so many charts. Of course, medical review of all charts would have been the true reference standard.

### Statistical analysis

From contingency tables, indicators of diagnostic accuracy, sensitivity, specificity, negative and positive predictive values, and positive and negative likelihood ratios were calculated along with an exact 95% confidence interval, using SAS 9.4 software (SAS Institute, Cary, NC., USA). Briefly, sensitivity or true positive rate is the number of positive index test (true positive) out of all stays with a target condition as defined by the standard procedure (true positive plus false negative). Specificity also called the true negative rate is the number of negative index test (true negative) that are identified as negative by the standard procedure (true negative plus false positive).

## Results

### Study population

Between 01/01/2014 and 12/31/2014 at Rennes University Hospital there were 49,792 emergency ward records and 22,400 hospital stays for adult patients. Out of these, we identified 16,012 hospital stays for adult patients with a hospital admission through emergency ward. Among these, the mean (SD) age was 61.7 (22.7) years and 52.2% were men.

### Index test

Across 16,012 hospital stays, the previously published algorithm [5] based on the hospital discharge main diagnosis, identified 637 bleeding events (Table 1). There were 293 intracranial hemorrhages (46%), 197 gastrointestinal hemorrhages (31%) and 147 other bleeding events (23%).

**Table 1:** Discharge diagnosis ICD-10 codes for 637 events identified by index test [5]

Type	Code	Label	Percent	Count
Intracranial hemorrhage	I61	Non-traumatic intra-cerebral hemorrhage	45.7	134
	S06	Intracranial injury	34.8	102
	I60	Non-traumatic subarachnoid hemorrhage	15.0	44
	I62	Other and unspecified non-traumatic intracranial hemorrhage	4.43	13
Gastrointestinal hemorrhage	K92	Other diseases of the digestive system	39.1	77
	K62	Other diseases of the anus and rectum	35.0	69
	K26	Duodenal ulcer	12.2	24
	K25	Gastric ulcer	11.7	23
	K28	Gastrojejunal ulcer	1.01	2
	K27	Peptic ulcer, site unspecified	0.51	1
	K29	Gastritis and duodenitis	0.51	1
Other bleeding	R04	Hemorrhage in the respiratory tract	48.9	72
	R31	Hematuria	26.5	39
	D62	Acute post-hemorrhagic anemia	10.9	16
	M25	Other joint disorder, not elsewhere classified	5.44	8
	H11	Ocular hemorrhage	1.36	2
	K66	Other disorders of the peritoneum	1.36	2
	N02	Recurrent and persistent hematuria	1.36	2
	N95	Menopausal and other peri-menopausal disorders	1.36	2
	R58	Hemorrhage, not elsewhere classified	1.36	2
	H92	Otalgia and effusion from the ear	0.68	1
	J94	Other pleural conditions	0.68	1

### Standard procedure

The automated first step identified 1959 records from the 16,012 eligible hospital stays, of which 736 were classified as major bleeding events by a medical expert review. All other stays (*n* = 15,276) were considered as without the target condition (1223 classified as such by medical expert review and 14,053 discarded by the automated first step).

### Main outcomes

**Fig. 1:**
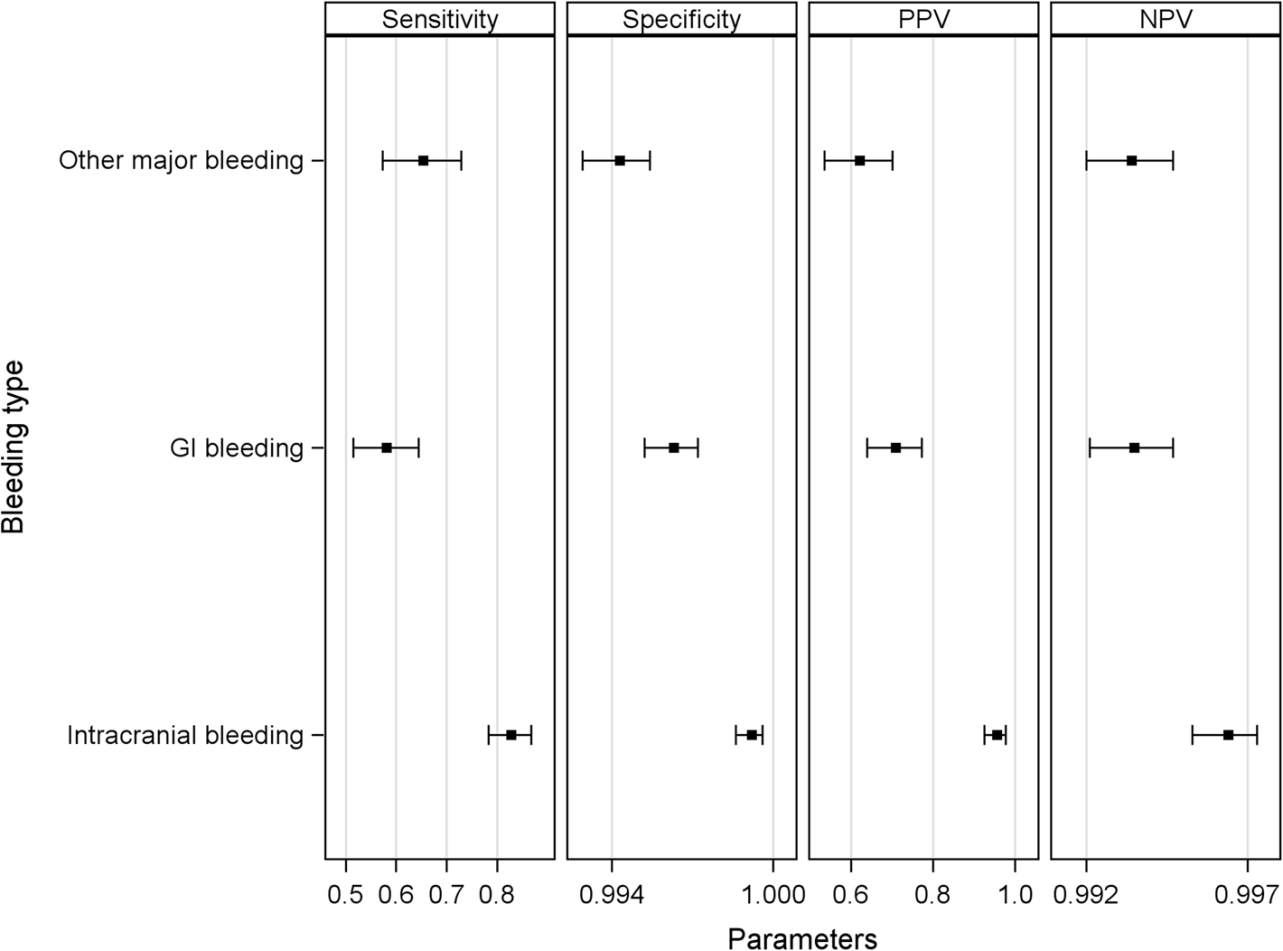
Accuracy of the previously used [5] ICD-10-based algorithm according to type of major bleeding**.** PPV and NPV denote positive and negative predictive values respectively

From the contingency table (Table 2) we derived the diagnostic performances (Table 3 and Fig. 1): there were 482 true positive index test results (positive index test - either ICH, GI or other bleeding according to the ICD-10 based algorithm - among those stays classified as having the target condition by the standard procedure), 15,121 true negative (negative index test among those stays classified as not having the target condition by the standard procedure), 155 false positive (positive index test among those stays classified as not having the target condition by the standard procedure) and 254 false negative (negative index test among those stays classified as having the target condition by the standard procedure); sensitivity (TP/TP + FN) and specificity (TN/TN + FP) varied across types of major bleeding, with the highest values for intracranial hemorrhage.

**Table 2:** Contingency table by type of hemorrhage

	Standard procedure		
	Positive	Negative	Total
Index test			
ICH	281	12	293
GI	141	56	197
Other	60	87	147
Negative	254	15,121	15,375
Total	736	15,276	16,012

The index test is an ICD-10-based algorithm (previously published [5])

*ICH* denotes intracranial hemorrhage, *GI* Gastrointestinal

**Table 3:** Accuracy of a previously used [5] ICD-10-based algorithm for major bleeding

N	True positive	True negative	False positive	False negative	Sensitivity % (95% CI)	Specificity % (95% CI)	PPV %(95% CI)	NPV %(95% CI)	Positive LR % (95% CI)	Negative LR % (95% CI)
16,012	482	15,121	155	254	65.5 (61.9 to 68.9)	99.0 (98.8 to 99.1)	75.7 (72.1 to 78.9)	98.3 (98.1 to 98.5)	64.5 (21.8 to ∞)	0.35 (0.00 to 2.08)

*PPV* and *NPV* denote positive and negative predictive values respectively, *LR* denotes likelihood ratio, *CI* denotes Clopper-Pearson’s (exact) confidence interval

### Description of false positives

Out of 155 hospital stays, 141 (91%) were identified by the automated first step, thereby indicating there was potentially a hemorrhage, but seriousness was ruled out by the medical expert review. It can be noted that these cases were mainly “other” bleeding events (62%) and gastrointestinal bleeding (37.4%); only 8 cases had ICD-10 codes for intracranial bleeding, 3 of them had code S06.5 (traumatic subdural hemorrhage) and another code S06.6 (traumatic subarachnoid hemorrhage). For the remaining 14 hospital stays, the ICD-10-based algorithm identified 3 “other” bleeding events, 6 gastrointestinal bleedings and 5 intracranial bleedings. Additional file 3 shows the diagnosis as coded in emergency ward and the main discharge diagnosis in order address the question of in-hospital bleeding as opposed to bleeding as the motive for referral, or no bleeding at all for these stays. For all intracranial bleedings, symptoms as coded in emergency wards were consistent with the main discharge diagnosis; this was not the case for gastrointestinal bleeding and other bleeding events where in-hospital bleeding may have occurred.

### Description of false negatives

Diagnoses (ICD-10 codes) as coded in emergency ward are shown in Table 4. Most of them (18 codes, totalizing 107 stays, 42%) were codes used by the ICD-10-based algorithm. Additional file 4 shows the main discharge diagnoses retained for these 107 stays: the codes were mostly based on etiology. The main discharge diagnosis codes mostly (75%) related to four chapters of the ICD-10 classification: S (injury, *n* = 94, 37%), D (*n* = 34, 13.4%, mostly diseases of the blood rather than neoplasms), I (*n* = 34, 13.4%), and K (*n* = 30, 11.8%).

**Table 4:** The most cited diagnoses (> 2%) in emergency ward out of 254 false negatives on the index test (an ICD-10-based algorithm, previously used [5])

Codes	Label	Percent	Count
*K62.5*	Hemorrhage of the anus and rectum	10.2	26
D64.9	Anemia, unspecified	7.87	20
R57.1	Hypovolemic shock	6.29	16
S39.0	Injury of muscles, fascia and tendon of abdomen, lower back and pelvis	5.90	15
*K92.0*	Hematemesis	5.51	14
*I61.9*	Nontraumatic intracerebral hemorrhage, unspecified	4.72	12
S00.0	Superficial injury of the scalp	3.15	8
S06.3	Focal traumatic brain injury	3.15	8
K66.1	Hemoperitoneum	2.36	6
K92.1	Melena	2.36	6
*R31*	Hematuria	2.36	6
T81.0	Complications of procedures, not elsewhere classified	2.36	6

Codes used by the ICD-10-based algorithm (but on discharge diagnoses) are in italics

## Discussion

First, we observed overall low sensitivity (65%); a third of the major bleeding events identified by our standard procedure were not detected by the ICD-10-based algorithm (false negatives). Second, we highlighted differential sensitivity and specificity across types of bleeding, with the highest values for intracranial hemorrhage. One hundred fifty-five cases out of 637 (24%) were false positives, with a large majority being non-serious, involving mostly “other” bleeding and GI bleeding events.

Major bleeding is an adverse event common to all types of antithrombotic drugs. An automated approach to identifying major bleeding in real time would be particularly useful. Indeed it would enable continuous monitoring and early signal detection in the area of pharmacovigilance. However, accuracy in coding for major bleeding is required to yield trustworthy results on the basis of hospital databases. To date, validation studies on ICD-10 code-based algorithms are scarce and focused on positive predictive values: only one study [9] identified major bleeding from emergency ward discharges using 35 ICD-10 codes for ICH or GI bleeding; a random sample was independently reviewed by two trained chart reviewers to validate the diagnosis, but no criteria to define major bleeding were applied, except for the location; the analysis showed an overall good positive predictive value of 88% (95%CI 83 to 91), with better estimates for ICH (90%) than for upper GI bleeding (74%). It can be noted that other forms of major bleeding events were not studied. These results based on emergency ward discharges were similar to others from studies using hospital discharge records (ICD-10 codes): Kokotailo et al. reported a positive predictive value of 98 and 91% for ICH and subarachnoid hemorrhage respectively [10]. Cunningham et al. showed that an algorithm identifying bleeding-related hospitalizations from the primary discharge diagnosis had a positive predictive value of between 89 and 99% in distinguishing specific bleeding sites [11]. The accuracy of upper GI bleeding codes in one Dutch administrative database using the ICD-10 coding system showed a positive predictive value of 77% [12]. In an assessment of four different coding systems in different European countries, it was concluded that positive predictive value is associated not only with the code itself, but is also with the way the code is used [12]; France was not part of this study. In France, main discharge diagnoses may not reflect the motive for referral, but rather what most impacts hospital resources; indeed the PMSI database has a primarily financial objective, not an epidemiological point of view. Lastly, Delate et al. recommended a manual chart review to validate warfarin-related bleeding events from administrative data [13]. Ruigomez et al. also advocated additional information to prevent misclassification as regards major GI or urogenital bleeding events [14]. Our findings are in line with these previous results and have highlighted differential measures of accuracy between ICH and GI. When evaluating anticoagulant safety profiles, it would be wise to perform separate analyses according to these outcomes. It is well known that non-differential misclassification biases the risk towards the null, but it can be noted that NOAC trials reported a lower risk of ICH but no significant decrease of GI bleeding; hence differential positive predictive values could be problematic when evaluating overall safety profiles.

The reported low sensitivity is a concern. This result might reflect a discrepancy between the motive for referral and the discharge coding (false negatives); it is worth noting that the PMSI does not collect emergency ward data; in any case, emergency ward data is thought to be unreliable with considerable inconsistency as a result of a lack of standardization. Only primary hospital discharge diagnoses were used in the index test to define major bleeding [5]. The consequence when using this algorithm (index test) will be an underestimation of the incidence of major bleeding. It has already been observed that when the code is listed as the most likely diagnosis or the admission diagnosis, a true bleeding event has occurred 96% of the time [15].

False positives from the algorithm (index test) were mostly non serious gastrointestinal bleeding or non serious other bleeding events. The point here is whether the algorithm catches in-hospital serious bleeding. This is important to consider when the question focuses on serious bleeding as a reason for hospital referral, which means bleeding potentially related to drug delivered on an ambulatory basis. Including in the analysis in-hospital serious bleeding might biased the results because at this time patients may not be exposed to the drug they were prescribed before hospital entry.

Our study has several strengths. Our sample size is by far the largest among studies testing the validity of ICD codes. We reviewed a consecutive set of charts irrespective of the ICD-10 coding allocated. Using negative controls, we calculated the sensitivity. In contrast, previous studies have been published without negative controls and have only reported positive predictive values. The medical chart review was blinded to the discharge diagnoses.

Our study also has several limitations. Firstly, the study was carried out by one medical expert reviewer in a single center. However, the reviewer followed objective criteria to determine the presence of major bleeding. On the other hand, inter-rater variability related to different level of expertise would have been an issue. Secondly, our definition for major bleeding was conservative, and it is likely that we underestimated the numbers of certain major bleeding events, especially with respect to the ISTH definition [16], which includes a drop in hemoglobin level of 20 g L^− 1^. The PMSI database does not include laboratory results. In addition, to take account of a drop in hemoglobin level, a reference level is required, which is not straightforward. Our definition applied only to hospital-based care. Therefore, data from individuals who do not seek medical attention or who are only seen in outpatient clinics or surgeries was not captured. For major bleeding, we thought this would not lead to a substantial bias, except for bleeding-related sudden death. Thirdly, we used a two-step approach as the standard procedure; an extensive chart review was simply unrealistic, because of the expense involved in reviewing so many charts. A previous pilot study has shown good sensitivity and specificity for the first automated step. Of course, medical review of all charts would have been the true reference standard.

## Conclusion

To conclude, the external validity of bleeding diagnostic codes has not been previously assessed in the French PMSI database. To the best of our knowledge, this is the first report. Our results showed overall low sensitivity, and, interestingly, different measures of accuracy across bleeding types. The results therefore provide support for specific data collection and a medical validation approach rather than an ICD-10-based algorithm for assessing the incidence of major bleeding.

## Supplementary information

**Additional file 1.** List of ICD-10 primary hospital diagnostic discharge previously published [5].

**Additional file 2.** Codes used by computerized requests made on electronic health records from emergency ward.

**Additional file 3.** Diagnosis as coded in emergency ward and main discharge diagnosis for 14 hospital stays (not identify as bleeding event by computerized request on electronic health records from emergency ward using hemorrhagic-related diagnostic codes and specific emergency therapies) out of 155 false positives.

**Additional file 4.** Main discharge diagnoses compared to emergency ward (EW) ICD-10 code for 107 stays where emergency ward diagnosis was coded as hemorrhage but not the main discharge diagnosis (false negative).

## Data Availability

The dataset used during the current study is available from the corresponding author on reasonable request.

## Abbreviations

ICD-10: International Classification of Disease, 10th edition
NPV: Negative predictive value
PMSI: French hospital database
PPV: Positive predictive value
SNDS: French National Health database
