# Supplementary material for: Sensitivity and specificity of an algorithm based on medico-administrative data to identify hospitalized patients with major bleeding presenting to an emergency department
Source: BMC Med Res Methodol. 2019 Oct 18;19:194. doi: 10.1186/s12874-019-0841-6 (PMC6798331; doi:10.1186/s12874-019-0841-6)
Supplement: Supplementary file 1 — Additional file 1. List of ICD-10 primary hospital diagnostic discharge previously published [5]. [file 12874_2019_841_MOESM1_ESM.pdf]

List of ICD-10 primary hospital diagnostic discharge previously published [5]

Type	ICD-10	Descriptions
Intracranial bleeding	I60	Nontraumatic subarachnoid hemorrhage
	I61	Nontraumatic intracerebral hemorrhage
	I62	Other and unspecified non-traumatic intracranial hemorrhage
	S06.3	Focal brain injury
	S06.4	Epidural hemorrhage
	S06.5	Traumatic subdural hemorrhage
	S06.6	Traumatic subarachnoid hemorrhage
Gastrointestinal bleeding	I85.0	Esophageal varices
	K25.0/2/4/6	Gastric ulcer
	K26.0/2/4/6	Duodenal ulcer
	K27.0/2/4/6	Peptic ulcer, site unspecified
	K28.0/2/4/6	Gastrojejunal ulcer
	K29.0	Gastritis and duodenitis
	K62.5	Hemorrhage of anus and rectum
	K92.0	Hematemesis
	K92.1	Melena
	K92.2	Gastrointestinal hemorrhage, unspecified
Other bleeding	D62	Acute posthemorrhagic anemia
	H11.3	Conjunctival hemorrhage
	H35.6	Retinal hemorrhage
	H43.1	Vitreous hemorrhage
	H45.0	Vitreous hemorrhage in disease classified elsewhere
	H92.2	Otorrhagia
	J94.2	Hemothorax
	K66.1	Hemoperitoneum
	M25.0	Hemarthrosis
	N02	Recurrent and persistent hematuria
	N92.0/1	Excessive and frequent menstruation
	N92.4	Excessive bleeding in the premenopausal period
	N93.8	Other specified abnormal uterine and vaginal bleeding
	N93.9	Abnormal uterine and vaginal bleeding, unspecified
	N95.0	Postmenopausal bleeding
	R04.0	Epistaxis
	R04.1	Hemorrhage from throat
	R04.2	Hemoptysis
	R04.8	Hemorrhage from other sites in respiratory passages
	R04.9	Hemorrhage from respiratory passages, unspecified
	R31	Hematuria
	R58	Hemorrhage, not elsewhere classified

* 0 = acute with hemorrhage, 2 = acute with hemorrhage and perforation, 4 = chronic or unspecified with hemorrhage, 6 = chronic or unspecified with hemorrhage and perforation
