# Supplementary material for: Sensitivity and specificity of an algorithm based on medico-administrative data to identify hospitalized patients with major bleeding presenting to an emergency department
Source: BMC Med Res Methodol. 2019 Oct 18;19:194. doi: 10.1186/s12874-019-0841-6 (PMC6798331; doi:10.1186/s12874-019-0841-6)
Supplement: Supplementary file 2 — Additional file 2. Codes used by computerized requests made on electronic health records from emergency ward. [file 12874_2019_841_MOESM2_ESM.pdf]

Codes used by computerized requests made on electronic health records from emergency ward

ICD-10	Descriptions
D50.9	Iron deficiency anemia, unspecified
D51.9	Vitamin B12 deficiency anemia, unspecified
D52.9	Folate deficiency anemia, unspecified
D62	Acute post-hemorrhagic anemia
D64.9	Anemia, unspecified
I31.2	Hemopericardium, not elsewhere classified
I60	Nontraumatic subarachnoid hemorrhage
I61	Nontraumatic intracerebral hemorrhage
I62	Other and unspecified non-traumatic intracranial hemorrhage
I98.3	Esophageal varices with bleeding in disease classified elsewhere
J94.2	Hemothorax
K22.6	gastro-esophageal laceration-hemorrhage syndrome
K25.%	Gastric ulcer
K26.%	Duodenal ulcer
K62.5	Hemorrhage of anus and rectum
K66.1	Hemoperitoneum
K92.0	Hematemesis
K92.1	Melena
K92.2	Gastrointestinal hemorrhage, unspecified
M25.0	Hemarthrosis
N93.9	Abnormal uterine and vaginal bleeding, unspecified
R04.0	Epistaxis
R04.1	Hemorrhage from throat
R04.2	Hemoptysis
R31	Hematuria
R57. 1	Hypovolemic shock
R58	Hemorrhage, not elsewhere classified
S01.0	Open wound of scalp
S06.0	Concussion
S06.2	Diffuse traumatic brain injury
S06.3	Focal brain injury
S06.4	Epidural hemorrhage
S06.5	Traumatic subdural hemorrhage
S06.6	Traumatic subarachnoid hemorrhage
S27.1	Traumatic hemothorax
S27.8	Injury of other specified intra-thoracic organs
S36.0	Injury of spleen
S39.0	Injury of muscle and tendon of abdomen, lower back and pelvis
T06.5	Injury of intra-thoracic organs with intra-abdominal and pelvic organs
T45.5	Poisoning by, adverse effect of and underdosing of anticoagulants and antithrombotic drugs
T79.2	Traumatic secondary and recurrent hemorrhage
T79.6	Traumatic ischemia of muscle
Y44.2	Agents primarily affecting blood constituents: anticoagulants
Y44.4	Agents primarily affecting blood constituents: antithrombotic drugs
