# Supplementary material for: Sensitivity and specificity of an algorithm based on medico-administrative data to identify hospitalized patients with major bleeding presenting to an emergency department
Source: BMC Med Res Methodol. 2019 Oct 18;19:194. doi: 10.1186/s12874-019-0841-6 (PMC6798331; doi:10.1186/s12874-019-0841-6)
Supplement: Supplementary file 3 — Additional file 3. Diagnosis as coded in emergency ward and main discharge diagnosis for 14 hospital stays (not identify as bleeding event by computerized request on electronic health records from emergency ward using hemorrhagic-related diagnostic codes and specific emergency therapies) out of 155 false positives. [file 12874_2019_841_MOESM3_ESM.pdf]

Diagnosis as coded in emergency ward and main discharge diagnosis for 14 hospital stays (not identify as bleeding event by computerized request on electronic health records from emergency ward using hemorrhagic-related diagnostic codes and specific emergency therapies) out of 155 false positives

Hospital discharge main diagnosis			Emergency ward diagnosis		
ICD-10 code	Label	Count	ICD-10 code	Label	Count
I60.2/.6	Nontraumatic subarachnoid hemorrhage	2	I72.9	Aneurysm of unspecified site	2
I60.8	Other nontraumatic subarachnoid hemorrhage	1	R51	Headache	1
J94.2	Hemothorax	1	J93.9	Pneumothorax, unspecified	1
K26.4	Duodenal ulcer chronic or unspecified with hemorrhage	1	K31.7	Polyp of stomach and duodenum	1
K62.5	Hemorrhage of anus and rectum	1	A41.9	Sepsis, unspecified organism	1
K92.0	Hematemesis	3	F32.9	Major depressive disorder, single episode, unspecified	1
			R41.0	Disorientation, unspecified	1
			F10.0	Mental and behavioral disorders related to alcohol use	1
K92.1	Melena	1	G35	Multiple sclerosis	1
R04.2	Hemoptysis	1	J45.9	Other and unspecified asthma	1
R31	Hematuria	1	E87.1	Hypo-osmolality and hyponatremia	1
S06.5	Traumatic subdural hemorrhage	2	G45.9	Transient cerebral ischemic attack, unspecified	1
			S42.2	Fracture of upper end of humerus	1
