# Supplementary material for: Sensitivity and specificity of an algorithm based on medico-administrative data to identify hospitalized patients with major bleeding presenting to an emergency department
Source: BMC Med Res Methodol. 2019 Oct 18;19:194. doi: 10.1186/s12874-019-0841-6 (PMC6798331; doi:10.1186/s12874-019-0841-6)
Supplement: Supplementary file 4 — Additional file 4. Main discharge diagnoses compared to emergency ward (EW) ICD-10 code for 107 stays where emergency ward diagnosis was coded as hemorrhage but not the main discharge diagnosis (false negative). [file 12874_2019_841_MOESM4_ESM.pdf]

Main discharge diagnoses compared to emergency ward (EW) ICD-10 code for 107 stays where emergency ward diagnosis was coded as hemorrhage but not the main discharge diagnosis (false negative)

Emergency ward diagnoses			Main discharge diagnoses	
Code	Label		Code	Label (number)
D62	Acute posthemorrhagic anemia	2	C787	Secondary malignant neoplasm of liver and intrahepatic bile duct
D62			S701	Contusion of thigh
I609	Nontraumatic subarachnoid hemorrhage	2	G459	Transient cerebral ischemic attack, unspecified
I609			R51	Headache
I619	Nontraumatic intracerebral hemorrhage	12	C833	Diffuse large B-cell lymphoma
I619			D33	Benign neoplasm of brain (3)
I619			G458	Other transient cerebral ischemic attacks
I619			G911	Obstructive hydrocephalus (2)
I619			I63	Cerebral infarction (2)
I619			S068	Other specified intracranial injuries (3)
I850	Esophageal varices with bleeding	3	I983	Esophageal varices with bleeding in disease classified elsewhere (3)
K250	Acute gastric ulcer with hemorrhage	2	K221	Ulcer of esophagus
K250			K318	Other specified diseases of stomach and duodenum
K260	Acute duodenal ulcer with hemorrhage		I728	Aneurysm of other specified arteries
K625	Hemorrhage of anus and rectum	26	C	Malignant neoplasm of rectosigmoid junction (2), cecum (1), rectum (2)
K625			D	Carcinoma in situ of colon (1), Neoplasm of uncertain behavior of colon (1), Refractory anemia (1)
K625			E559	Vitamin D deficiency, unspecified
K625			I713	Abdominal aortic aneurysm, ruptured
K625			I983	Esophageal varices with bleeding related to disease classified elsewhere
K625			K5	Acute vascular disorders of intestine (2), Diverticular disease of large intestine without perforation or abscess (4)
K625			K6	Ulcer of anus and rectum (2), Fourth degree hemorrhoids (1)
K625			K7	Alcoholic cirrhosis of liver 1), Acute and subacute hepatic failure (1)
K625			R57	Other shock (1), Hypovolemic shock (1)
K625			S366	Injury of rectum
K625			Z433	Encounter for attention to colostomy
K661	Hemoperitoneum	4	R18	Ascites
K661			S366	Injury of rectum (1), other intra-abdominal organs (3), spleen (1)

Emergency ward diagnoses			Main discharge diagnoses	
Code	Label		Code	Label (number)
K920	Hematemesis	14	C	Malignant neoplasm of tonsil (1), of fundus of stomach (1), MALT-lymphoma (1)
K920			I713	Abdominal aortic aneurysm, ruptured
K920			I983	Esophageal varices with bleeding in disease classified elsewhere (7)
K920			K221	Ulcer of esophagus (1), Gastro-esophageal laceration-hemorrhage syndrome (1)
K920			N10	Acute pyelonephritis
K921	Melena	6	C160	Malignant neoplasm of cardia
K921			D508	Other iron deficiency anemias
K921			I983	Esophageal varices with bleeding in disease classified elsewhere
K921			K2	Ulcer of esophagus (1), Acute duodenal ulcer without hemorrhage or perforation (1)
K921			K573	Diverticular disease of large intestine without perforation or abscess
R040	Epistaxis	4	D619	Aplastic anemia, unspecified (1), Drug-induced aplastic anemia (2)
R040			N185	Chronic kidney disease, stage 5
R041	Hemorrhage from throat	3	D695	Secondary thrombocytopenia
R041			J358	Other chronic diseases of tonsils and adenoids
R041			S008	Superficial injury of other parts of head
R042	Hemoptysis	2	J201	Acute bronchitis due to Hemophilus influenzae
R042			J701	Chronic and other pulmonary manifestations due to radiation
R31	Hematuria	6	C679	Malignant neoplasm of bladder, unspecified
R31			D500	Iron deficiency anemia secondary to blood loss (chronic)
R31			N3	Irradiation cystitis (1), Other specified disorders of urethra (1)
R31			N40	Benign prostatic hyperplasia
R31			R33	Retention of urine
S063	Focal traumatic brain injury	8	D330	Benign neoplasm of brain, supratentorial
S063			M808	Other osteoporosis with current pathological fracture, vertebra(e)
S063			S06	Diffuse traumatic brain injury (2), Other specified intracranial injuries (2)
S063			S720	Fracture of head and neck of femur
S063			S836	Sprain of the superior tibiofibular joint and ligament
S064	Epidural hemorrhage	2	G951	Vascular myelopathies
S064			S062	Diffuse traumatic brain injury
S065	Traumatic subdural hemorrhage	5	S0	Fracture of base of skull (1), Concussion (1), Other specified intracranial injuries (1)
S065			S270	Traumatic pneumothorax

Emergency ward diagnoses			Main discharge diagnoses	
Code	Label		Code	Label (number)
S065			S510	Open wound of elbow
S066	Traumatic subarachnoid hemorrhage	3	F103	Alcohol related disorders (with withdrawal seizure)
S066			S131	Subluxation and dislocation of cervical vertebrae
S066			S021	Fracture of base of skull
